# Recycling Aggregates for Self-Compacting Concrete Production: A Feasible Option

**DOI:** 10.3390/ma13040868

**Published:** 2020-02-14

**Authors:** Rebeca Martínez-García, M. Ignacio Guerra-Romero, Julia M. Morán-del Pozo, Jorge de Brito, Andrés Juan-Valdés

**Affiliations:** 1 Department of Mining Technology, Topography and Structures, University of León, Campus de Vegazana s/n, 24071 León, Spain; rmartg@unileon.es; 2 Department of Agricultural Engineering and Sciences, University of León, Avenida de Portugal 41, 24071 Léon, Spain; ignacio.guerra@unileon.es (M.I.G.-R.); julia.moran@unileon.es (J.M.M.-d.P.); 3 Department of Civil Engineering, Architecture and Georresources, IST-Universidade de Lisboa, Av. Rovisco Pais, 1049-001 Lisbon, Portugal

**Keywords:** self-compacting concrete, coarse recycled aggregate, sustainable concrete, construction and demolition waste management plant

## Abstract

The use of construction and demolition wastes (C&DW) is a trending future option for the sustainability of construction. In this context, a number of works deal with the use of recycled concrete aggregates to produce concrete for structural and non-structural purposes. Nowadays, an important number of C&DW management plants in the European Union (EU) and other countries have developed robust protocols to obtain high-quality coarse recycled aggregates that comply with different European standards in order to be used to produce new concrete. The development of self-compacting concrete (SCC) is another way to boost the sustainability of construction, due to the important reduction of energy employed. Using recycled aggregates is a relatively recent scientific area, however, studies on this material in the manufacture of self-compacting concrete have proven the feasibility thereof for conventional structural elements as well as high-performance and complex structural elements, densely reinforced structures, difficult-to-access formwork and difficult-to-vibrate elements. This paper presents an original study on the use of coarse recycled concrete aggregate (CRA) to obtain self-compacting concrete. Concrete with substitution ratios of 20%, 50% and 100% are compared with a control concrete. The purpose of this comparison is to check the influence of CRA on fresh SCC as well as its physical and mechanical properties. The parameters studied are material characterization, self-compactability, compressive strength, and tensile and flexural strength of the resulting concrete. The results conclude that it is feasible to use CRA for SCC production with minimal losses in the characteristics.

## 1. Introduction

The construction sector has seen an exponential growth in recent decades throughout the European Union (EU). This growth has led to an increase in the production of construction and demolition waste (C&DW). The developed countries are a major consumer and generator of waste. According to the European Statistics Office, Eurostat, every EU citizen produces an average of 2000 kg of waste per year (not including waste from mining, if mining waste were included, this figure would be 5000 kg/person/year) [1].

More than a third of all waste generated in the EU comes from the construction sector considering that, after water, aggregates are the raw material most heavily-consumed by man and it is mainly used in concrete, which means the valorisation of this type of waste needs to be promoted. This would reduce the extraction of natural aggregates and eliminate difficult-to-manage waste [2,3].

Government agencies, the scientific community and the population, in general, are becoming increasingly aware of the depletion of natural resources and advocating sustainable development, which leads to the need of recycling waste so that resources can be part of a circular economy and can be sustained from generation to generation. Everyone is aware of the need for combining economic development with sustainability and environmental protection.

It is in this context that new applications and developments for C&DW have been studied. The production of concrete with recycled aggregates has been one of the most often studied applications. It is true that some reliable studies currently support the use of C&DW in conventional concrete [4,5,6,7,8,9,10]. In addition, some studies have been published in recent years on the use of this waste in self-compacting concrete (SCC) [11,12,13,14,15,16,17,18,19,20,21,22,23,24,25,26,27].

In these studies, on the one hand, a reference (or control SCC) concrete mix with natural aggregates is designed and, on the other hand, different mixes of SCC with various incorporation ratios of coarse recycled concrete aggregate (CRA) are tested to compare with the control SCC. Most of the studies only replace the coarse fraction of the aggregate and only some of them replace the sand fine fraction. We have selected the studies in which the recycled aggregates come from old concrete structures as in our case, discarding other normally using ceramic materials.

Grdic et al. [28] designed three types of mixes with the incorporation of 0%, 50% and 100% of CRA. The type of cement used was CEM/II/B-M 42.5N maintaining a constant quantity of 409.6 kg/m^3^. The water absorption of the recycled aggregate was 5.08%, and the SP ratio was kept constant (0.7% relative to the weight of cement) and the water content was adjusted, increasing with the substitution ratio.

Safiuddin et al. [22] designed five types of mixes with substitutions of 0%, 30%, 50%, 70% and 100% of CNA with CRA. They employed CEM Type I, with a weight per m^3^ of 342 kg/m^3^. The W/C ratio and the content of SP are constant in all the mixes, 0.60 W/C and 1.50% respectively. The water absorption of the recycled aggregate used was 1.32%.

Tuyan et al. [29] design four types of mixes with substitutions of 0%, 20%, 40% and 60% of CNA with CRA. The cement was class C with a content of 315 kg/m^3^. Three W/C ratios are used, 0.43, 0.48 and 0.53. The SP content was adjusted in relation to the percentage of replacement from 0.95% to 1.97% of the cement weight. The water absorption of the recycled aggregate was 4.80%.

Modani et al. [30] studied six types of mixes with substitutions of 0%, 20%, 40%, 60%, 80% and 100% employing a Grade 53 cement with 348 kg/m^3^. The W/C ratio was 0.53. The SP was adjusted in relation to the replacement ratio from 1.3% to 1.58% of the cement weight. The water absorption of the recycled aggregate used was 5.64%.

Pereira de Oliveira et al. [21] designed four types of mixes with substitutions of 0%, 20%, 40% and 100% of CNA with CRA employing two types of recycled aggregates, using CEM I 42.5-R at 284.9 kg/m^3^. The W/C ratio was 0.56 and 0.57. The SP was adjusted in relation to the replacement ratio from 1.19% to 2.10% of the cement weight. The water absorption of the two used recycled aggregates was 4.10% and 4.05%.

As a general rule, a number of studies specify that the addition of recycled aggregates in small proportions (≤20%) in substitution of natural aggregates in concrete does not lower the performance of the resulting concrete. These studies have made it possible for these types of recycled materials to be included in standards and/or recommendations throughout the world. At a national level, Annex 15 “Recommendations for using recycled concrete” of the Spanish Structural Concrete Instruction EHE-08 [31] sets forth a few guidelines for its use: a) by limiting the content of coarse recycled aggregate (CRA) to 20% of the total weight of coarse aggregates, the final properties of recycled aggregates concrete are hardly affected and it allows experimental studies for substitution at higher ratios, b) not allowing the use of fine recycled aggregates irrespective of their nature, c) excluding the use of mixed recycled aggregates.

SCC is a high-performance concrete, the main characteristic of which is its fluid and viscous consistency allowing it to flow through densely reinforced structural elements without the addition of outside energy for compaction. Its composition is characterized by a high cement and fine particle content, a lower proportion of coarse aggregate and the use of next-generation additives [32].

The materials used to produce SCC are the same as for conventional concrete yet with some type of admixture, such as superplasticizer (SP) or viscosity modifiers, which are essential to avoid segregation and exudation of the mix. Special attention must be paid to the choice of materials in order to guarantee uniformity and consistency. This is complicated when using recycled aggregates for substitution purposes considering their great heterogeneity [32].

Some authors and studies recommend the use of Portland cement Type II (with a clinker content between 65% and 94%) for use in concrete with recycled aggregates to prevent the generation of alkali-aggregate reactions [33]. Other authors also recommend cement with a strength of no less than 42.5 MPa so that this characteristic is not a limiting factor. Moreover, the use of a sulphate-resistant cement seems interesting due to the possibility, according to some authors, of a reaction between the hydrated tricalcium aluminate of the hardened concrete and external sulphates, which leads to the formation of sulfoaluminate along with an increase in volume. Using sulphate-resistant cement would minimize this effect [10]. A number of studies ratify the higher absorption capacity of recycled aggregates at around 10% extra water and this is a premise to be considered. This higher demand for water is compensated by the use of superplasticizers [22,33,34,35,36,37].

Annex 17 “Recommendations for the use of SCC” of the Spanish Structural Concrete Instruction EHE-08 [31] provides a few guidelines: a) the maximum size of the aggregate shall be limited to 25 mm, b) the essential use of superplasticizers and/or viscosity modifiers in this type of concrete to guarantee self-compactability, c) it must comply with the project specifications, i.e., structural, operational and environmental requirements, d) the total fine particle content must be 450–600 kg/m^3^, the cement content 250–500 kg/m^3^, and the paste volume above 350 litres/m^3^.

The use of recycled sand is not allowed by the Spanish Instruction for Structural Concrete [31] even though some studies show that substitutions of up to 10% do not produce any significant variations in the characteristics of concrete [15,17,19]. Currently, Instruction EHE-08 allows the partial addition (<20% weight) of coarse recycled concrete aggregates as replacement of natural aggregates.

The aim of this research study is to characterize recycled aggregates and design the optimal and feasible content to produce SCC by substituting coarse aggregate fractions with recycled aggregates at 20%, 50% and 100%. The purpose of this comparison is to examine the influence of CRA on SCC concerning the fresh and physical and mechanical properties. Four types of concrete mixes were produced for this study (CC or control concrete, Mix 20, Mix 50, Mix 100). The characterization of the materials in the mix was studied, compactability tests were performed on the fresh state as well as tests in the hardened state: compressive, tensile and flexural strength.

The use of recycled aggregates in the production of self-compacting concrete has been gradually increasing due to the economic and environmental advantages derived from the fusion of the two techniques. On the one hand, there are the advantages of SCC: optimal compaction without vibration, fluidity, complex or specially reinforced works, increase in the quality of the finish in exposed concrete, and greater adherence. On the other, there are the advantages of recycled aggregates: less demand for extraction of natural aggregates from quarries, reduction of the pollution produced by C&DW, savings in transport costs, low energy consumption, less investment as a good quality material is produced at a low cost, and resources are optimised. However, information on the quality of SCC with RCA is still scarce. This study (as well as other similar ones) provides very useful information for the practical use in concrete production. One of the main novelties of our study is the use of real coarse recycled aggregates, obtained from a C&DW management plant, with standard treatment, unlike some other studies that use a specific recycled aggregate obtained from only one specific demolition, construction or maintaining work.

## 2. Materials and Methods

### 2.1. Materials

This research focused on the study of the mechanical and rheological properties of SCC produced with coarse recycled aggregates from crushed concrete elements designed as per the Okamura recommendations [38] as well as the minimum requirements of Instruction EHE-08 [31].

**Figure 1:**
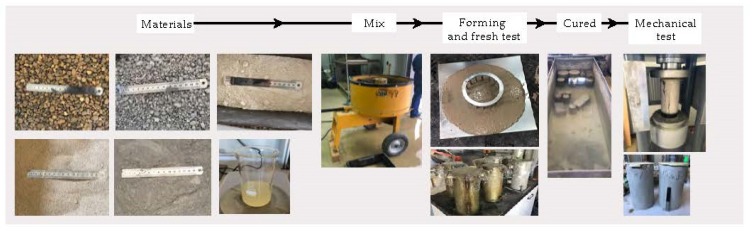
Research sequence.

The characteristics of the materials used in its production were carefully studied in the design of SCC as this has an immense impact on the behaviour of the mix. Figure 1 shows the study sequence.

#### 2.1.1. Paste

The paste for the concrete mixes was made with Portland CEM Type III/A 42.5 N/SR cement, which complies with the physical, chemical and mechanical requirements established in European standard EN 197-1:2013 [39]. The cement used, CEM type III, is cement with blast furnace slag addition. The content of clinker is 54% (EN-197 indicates that it must be 35–65%) and that of slag is 41% (the standard establishes 36–65% by weight), and it may contain up to a maximum of 5% of minor components, usually with gypsum as a setting regulator. Table 1 provides its chemical composition.

**Figure 2:**
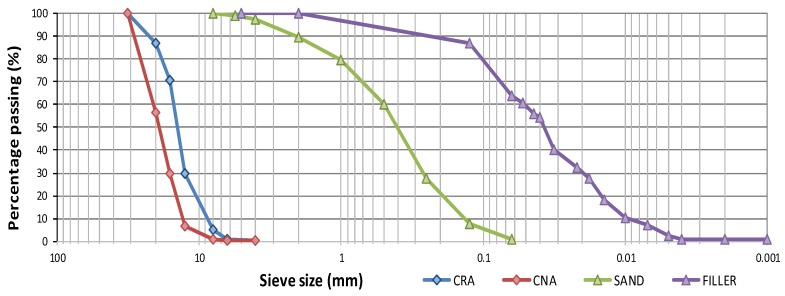
Particle size distribution of natural lime filler, natural sand, coarse natural aggregate (CNA) and coarse recycled aggregate (CRA).

Natural lime filler from a commercial plant was used as an addition. The chemical composition is shown in Table 2 and the particle size in Figure 2.

#### 2.1.2. Aggregates

**Figure 3:**
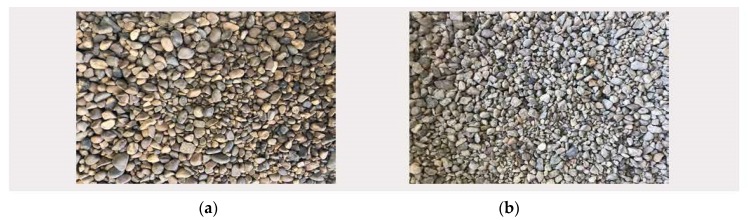
(**a**) Coarse natural aggregates, (**b**) Coarse recycled aggregates.

Three aggregates were used to produce concrete: 0–4 mm fine siliceous aggregate, 4–12.5 mm natural siliceous river gravel and 4–12.5 mm recycled aggregate from concrete elements. The characteristics of the aggregates used are specified below. Figure 3 shows the appearance of natural and recycled coarse aggregates. The fineness module is 5.35 for CNA, 4.7 for CRA, and for natural sand. The mean diameters are 5.91 mm for natural sand, 11.20 mm for CRA and 12.27 for CNA.

##### Composition and Characterization of the Recycled Aggregates

**Figure 4:**
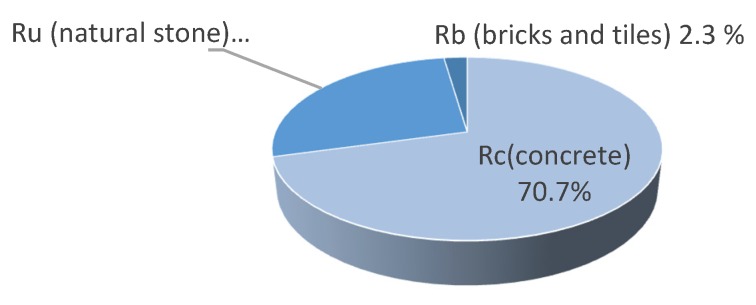
Coarse recycled aggregates composition according to EN 933-11:2009 [40].

The recycled aggregates were supplied by C&DW recycling plant. The physical and chemical characteristics established by EHE-08 were used to check the degree of compliance of the results with the acceptable limits (Table 3). Figure 4 shows the macroscopic composition of the aggregates, which are mostly made of concrete, stone, low content of ceramic material and a residual quantity of gypsum EN 933-11:2009 [40]. As concerns the morphology, it is irregular in shape with sharp edges and surface roughness.

The absorption of aggregate is slightly lower than the EHE-08 limit. This type of aggregate often shows high absorption values, but this specific aggregate is not affected by a higher absorption capacity due to the small quantity of ceramic material and attached mortar. Therefore, in principle, it does not need to be offset with a larger quantity of mixing water. Figure 2 shows the size distribution of the coarse recycled aggregates as per EN 933-3:2012 [42].

##### Composition and Characterization of the Natural Coarse and Fine Aggregates

The natural siliceous river coarse aggregate was supplied by a local company. It has a grain size of 4-12.5 mm. Table 4 shows its characteristics.

The quantitative analysis of the chemical composition of the natural coarse aggregates was carried out by means of X-ray fluorescence (XRF). Table 2 shows the results of the analysis, confirming the siliceous nature of the aggregates. Concerning the morphology, a visual inspection indicated it is more regular in shape with rounded edges and a smoother, more polished and impermeable surface than the recycled aggregate. Figure 2 shows the coarse natural aggregates size distribution.

The fine natural aggregates also have a siliceous nature with a grain size of 0-4 mm. The grain size curve is described in Figure 2. The quantitative analysis of the chemical composition of the fine natural aggregates is also carried out by X-ray fluorescence (XRF). Table 2 shows the results of the siliceous nature of this aggregate.

#### 2.1.3. Superplasticizer Additive

A third generation commercially available superplasticizer (SP), an aqueous-based modified polycarboxylate was used.

#### 2.1.4. Water

Finally, the mixing water used came from the city of León drinking water supply. It meets the standards established in EHE-08.

### 2.2. Compositions

SCC was designed considering the instructions found in EHE-08 [31] and following Okamura method’s procedures [32], modified as proposed by other authors. A cement content of 400 kg/m^3^ and a W/C ratio of 0.47 were used as indicated by the standard.

Concrete was mixed in a vertical axis planetary concrete mixer. Once mixing ended, the mix was poured into different moulds, according to the proposed test and then submerged in a curing chamber for 28 days.

A reference concrete or control mix was designed as well as mixes substituting 20%, 50% and 100% of coarse natural aggregates with coarse recycled aggregates (Table 5).

The amounts of materials used in the mix are shown in Table 6.

The base mix was modified by increasing the superplasticizer volume (SP:CC = 0.8%, Mix20 = 1%, Mix 50 = 1.2% and Mix 100 = 1.35%) to achieve the self-compactability parameters, finally determining an optimal quantity of admixture of 0.8% with respect to the weight of cement for the control concrete. The W/C ratio was maintained in the mixes, for comparability purposes.

Different batches have been done to test the rheological and mechanical characteristics of the mixes. Four different batches per mix (CC, Mix20, Mix50 and Mix100) were produced (1) for J-ring test, (2) for compressive strength, (3) for flexural strength, and (4) for tensile strength tests. The number of samples per batch was at least three, to ensure enough representativeness of the results.

### 2.3. Mixing Protocol

**Figure 5:**
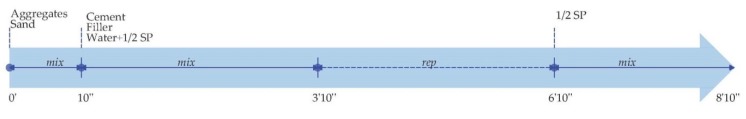
Mixing protocol.

The concrete mixing procedure used for all samples was based on standard EN 12390-2:2009 [45], following the steps shown in Figure 5.

Firstly, the mixer was moistened with water to prevent accumulation. The coarse natural and/or recycled aggregates were added from largest to smallest and later sand was added and then mixed for 10 s. The cement and filler were added and mixed for three minutes, slowly pouring in the mixing water with half of the superplasticizer, with time beginning upon completely pouring in the mixing water. The mix was left to stand for three minutes and was then mixed for another two minutes. The rest of the superplasticizer was poured homogeneously during no more than the first 10 s. The mixing was completed and testing began with fresh concrete.

To determine the self-compacting parameters prescribed, a flow test was done as per EN 12350-8:2011 [46], as well as a flow test using the J-ring test as per EN 12350-12:2011 [47] Both tests were performed immediately after mixing was finished.

Compressive strength was determined in the hardened state as per EN 12390-3:2009 [48] at 7, 14 and 28 days with cylindrical samples (300 × 150 mm).

Tensile and flexural strength were determined in the hardened state as per EN 12390-6:2010 [49] and EN 12390-5:2009 [50] respectively at 28 days old samples, 300 × 150 mm cylindrical samples were used for tensile strength and 100 × 100 × 400 mm prismatic samples for flexural strength.

## 3. Results and Discussion

### 3.1. Assessment of Coarse Recycled Aggregate

It is a concrete-sourced material that comes from C&DW of concrete elements with little rock, ceramic and gypsum impurities. As can be observed, the grain size of the recycled aggregates as well as of the natural aggregates is 4.5–12 mm, which exceeds the minimum size requirements for aggregates established by the Spanish code EHE-08. Although the minimum size of 4 mm is limiting, the grain size is believed to favour the self-compactability and workability of concrete adequate for these applications. Other studies often use grain sizes closer to 8–16 mm [11].

### 3.2. Composition

The amount of cement and W/C ratio limit values established by EHE-08 were complied with. According to the conclusions of other experimental studies [15,19], the adequate composition is somewhat enhanced and different from the one obtained by the strict use of the Okamura method [51]. Many of the tests consulted use cement contents ranging from 285 kg/m^3^ to 440 kg/m^3^ and compensating this range of values with more or less fine particles [11,19,26]. Other authors use 52.5 cement since its greater fineness helps with the workability and flow.

In the mixes proposed herein, the W/C ratio was maintained at 0.47 and no extra mixing water was added even though other authors correct this ratio as the proportion of recycled aggregates is increased, with up to 10% extra mixing water [21]. Other authors also use other valid techniques such as pre-saturating the aggregates [52]. Although the recycled aggregates used show a water absorption below the limits established by the standard, as shown in Table 3, it continues to be higher than the absorption of natural aggregate and, as has been proved, it was necessary to increase the percentage of superplasticizer based on the ratio of recycled aggregates. Other tests by other authors also consider this extra superplasticizer proportional to the increase in recycled aggregates [11,18].

### 3.3. Self-Compactability

**Figure 6:**
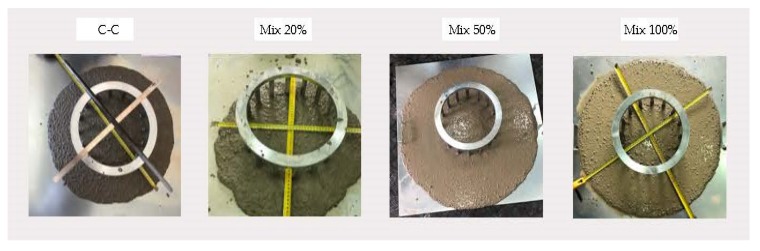
Japanese Ring test.

The mixes were repeated twice, and the slump flow was measured with the J-Ring test (Figure 6). The Japanese ring flow test, J-Ring, assesses the blocking resistance of SCC through reinforcement bars under free-flow conditions. Moreover, any segregation, exudation and a higher concentration of the coarse aggregates in the central area can be observed. The standards in effect for this test are EN 12350-8:2011 [46] and EN 12350-12:2011 [47]. All mixes comply with the reference values shown in Table 7, self-compactability criteria.

**Figure 7:**
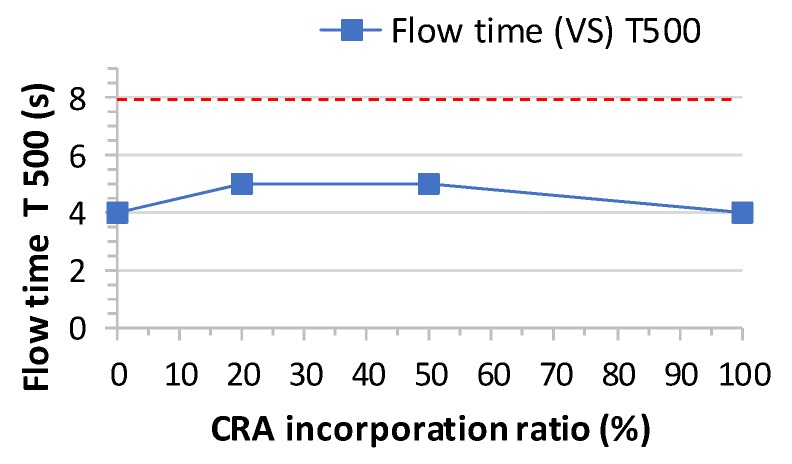
Flow time T_500_ test results.

**Figure 8:**
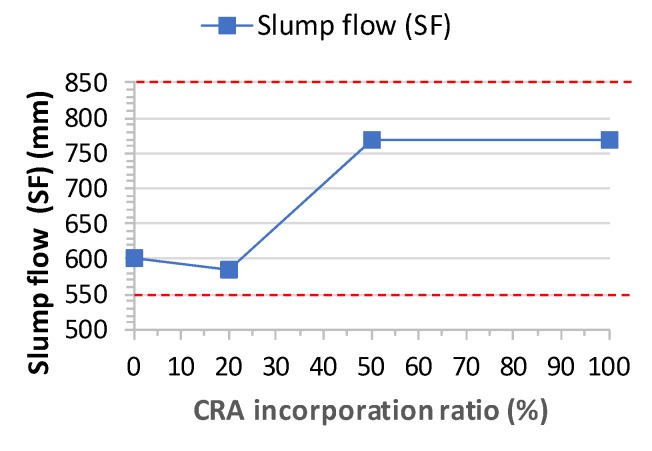
Slump flow test results.

**Figure 9:**
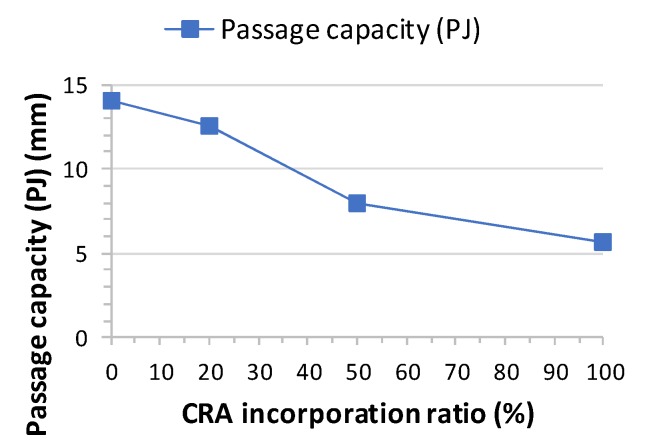
Passage capacity test results.

The slump flow time T_500_ results of the concrete designed with different CRA contents are shown in Table 7. The T_500_ slump flow time varies between 4 and 5 s (Figure 7). Figure 8 shows a comparison between slump flow and substitution of coarse aggregates. As per EHE-08 [31], EFNARC [53] and other standards and guides, it should be within ranges of 2 to 5 s. Therefore, the times achieved are within the acceptable ranges and recycled aggregates SCC meets the requirements of the various standards (Figure 9).

In terms of slump flow, a higher ratio of CRA leads to a larger final diameter and smaller passage capacity. The effect of CRA on the T_500_ slump flow time of the SCC should have been obvious for the 50% and 100% mixes, but they did not become more viscous with the increase in the percentage of superplasticizer. Visually, Mix 20 and Mix 50 reflected a good distribution of coarse aggregate and an absence of segregation and bleeding (Figure 6).

**Figure 10:**
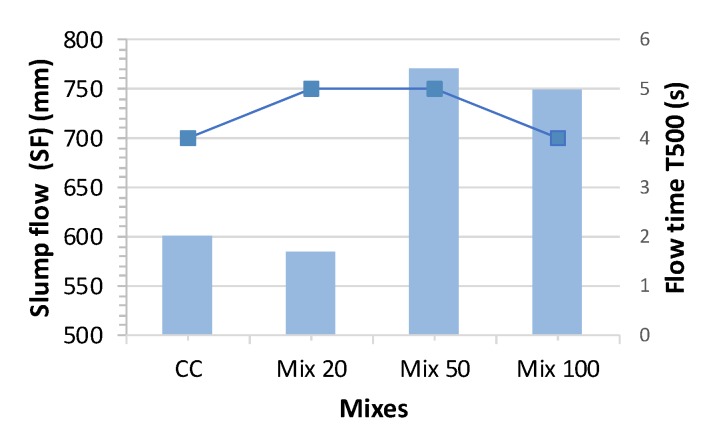
Slump flow time test results.

**Figure 11:**
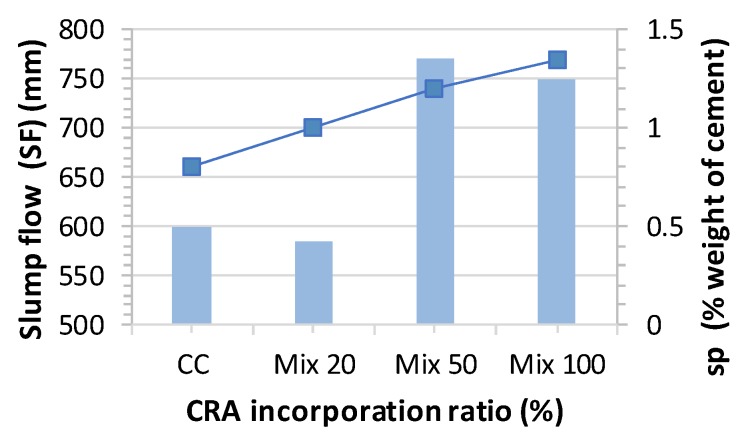
Slump flow and superplasticizer content.

Generally, slump flow decreases with the CRA incorporation ratio since more water is absorbed as the ratio increases. Some authors, such as Safiuddin et al. [22], Tuyan et al. [29] and Modani and Mohitkar [30], have found that the slump flow increases for relatively small CRA incorporation ratios (20–40%) and the slump flow decreases for higher ratios (70–100%) because the fine aggregates content tend to increase due to the fracture during mixing and the resulting higher water absorption. However, according to Pereira-de-Oliveira el al. [21], slump flow increases with CRA’s incorporation ratio given the gradual increase of SP content. This theory matches our results (Figure 10 and Figure 11).

Minimal variation was observed in the content of SP in the mixes at just 0.1% (relative to the weight of cement), which greatly influences the self-compactability of the mix. Readjusting the content of SP in some of the mixes could be considered: e.g., adjusting Mix 20 to 1.05–1.10% of SP and mix 100 to 1.325% of SP would be appropriate.

**Figure 12:**
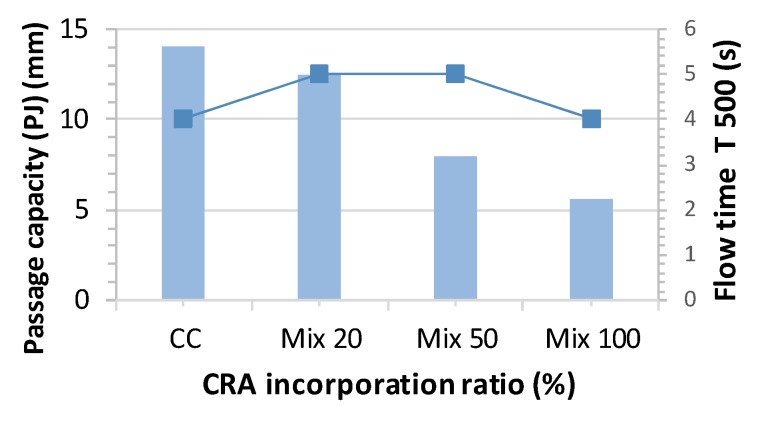
PJ and T_500_ results.

Figure 12 shows the flow time (T500) and the passage capacity (mm) of the different mixes in order to classify the self-compactability of concrete.

The classification obtained for concrete as per the criteria found in EHE-08, Annex 17 [31] and standard EN 12350-8:2011 [46] would be as follows:

- Based on the flow class: Class AC-E1,
- Based on the viscosity class: AC-V1,
- Based on the blocking resistance class: AC-RB2.

In general, class AC-E1 is the most adequate for most structural elements normally built, although it would be better to use a more fluid concrete such as type AC-E3 with 750 mm ≤ SF ≤ 850 mm for densely reinforced structures and formwork that is difficult to access or demands a long horizontal pouring displacement [31].

### 3.4. Compressive Strength

**Figure 13:**
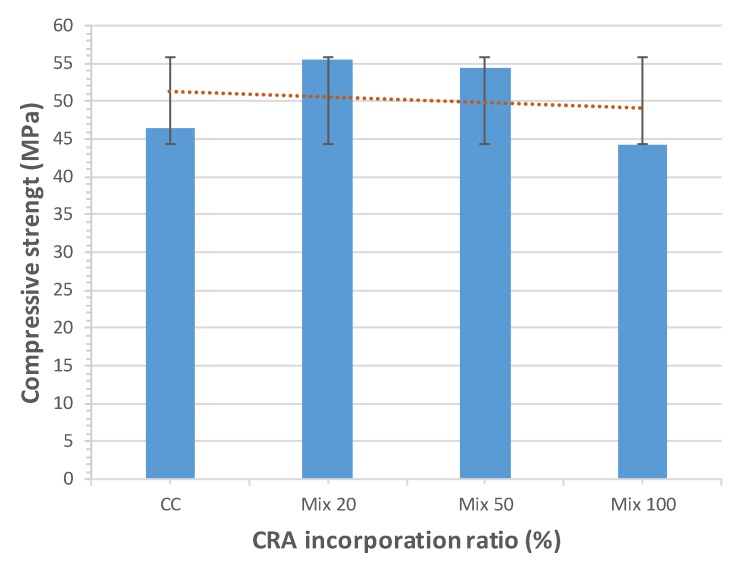
28-day compressive strength results.

The compressive strength test was determined as per standard EN-12390-3 [48]. The results are shown in Figure 13. In terms of compressive strength, the recycled aggregates studied herein led to a noticeable difference. The compressive strength of Mix 20 and Mix 50 is higher than that of CC. For Mix 20 it is 55.58 MPa, which is 20% higher than that of CC (46.36 MPa). The average value for Mix 50 is 18% higher than for CC and Mix 100′s is 5% lower than CC. This nonconformity may be due to the fact that the recycled aggregates in the composition, as shown in Table 3, have a high content of concrete that may positively influence the mechanical behaviour. A well-known effect is that the compressive strength decreases as the W/C ratio grows. In this study, the W/C ratio remains constant for all mixes, but the content of SP increases with the incorporation of CRA. This may explain why the compressive strength drops for Mix 100.

Grdic et al. [28] concluded that compressive strength decreases slightly as replacement increases (3.88% for 50% CRA and 8.55% for 100% CRA). The explanation for this reduction in strength can be found in the microstructure, i.e., the irregular composition of the CRA (formed by natural aggregate and cement paste) which provokes this slightly reduction.

Tuyan et al. [29] found that compressive strength increases as the W/C ratio decreases. Compressive strength increases slightly in mixes containing up to 40% CRA, about 5% with respect to the control mix. For higher replacement ratios (60%), compressive strength decreases. This is due to the microstructure, pores and cracks formed in the interfacial transition zones (ITZ) between the CRA and the paste slightly weakening the overall structure of the SCC.

The study of Modani et al. [30] concluded that, as the percentage of CRA increases, compressive strength decreases. This reduction is justified by the mortar and impurities adhering to CRA, creating areas of weakness in the SCC. For the 40% CRA mix, the loss was 5.80% compared to the control concrete. The mix containing the highest amount of CRA did not maintain the gain in strength over time due to the presence of unhydrated cement in CRA.

Pereira de Oliveira et al. [21] established that the replacement of CRA does not significantly influence the mechanical behaviour of the SCC, and finds a 5% difference between the control SCC and the 100% replacement mix.

**Figure 14:**
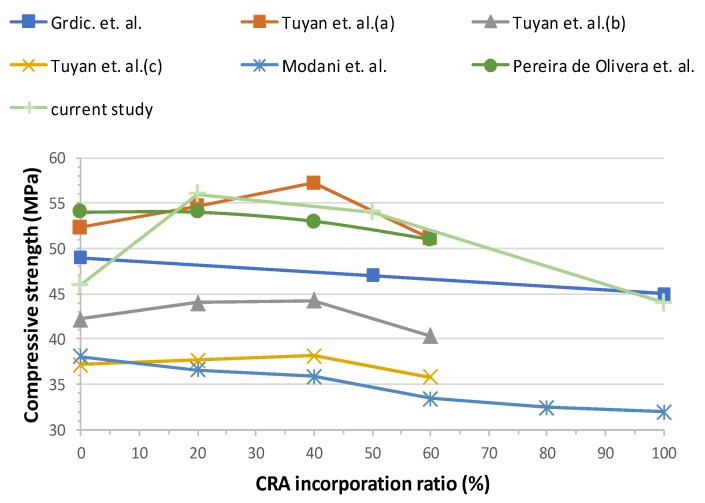
28-day compressive strength results overview.

The comparison with previous studies showed that, for a 20% substitution ratio, our results share the same trend and range of Tuyan et al. (a) [29] and Pereira de Oliveira et al. [21], with values between 54 MPa and 56 MPa. The trend line is very similar in all studies, i.e., up to 40–50% substitution, the compressive strength slightly increases relative to the control concrete and, for higher values of substitution, the compressive strength decreases. Figure 14 shows the comparison of our study with the results obtained by other authors. For total substitution (100%), our results match the figures obtained by Modani et al. [30]. Most of the authors studied [22,28,29,30] generally conclude that compressive strength slightly decreases with the CRA incorporation ratio. Some authors [21] conclude that compressive strength does not vary significantly with the incorporation of CRA because of the high volume of paste involved.

With these compressive strength values, it is perfectly possible to reach f_ck_ of 40 MPa, coherent with the W/C ratio and the amount of cement used.

### 3.5. Tensile Strength

**Figure 15:**
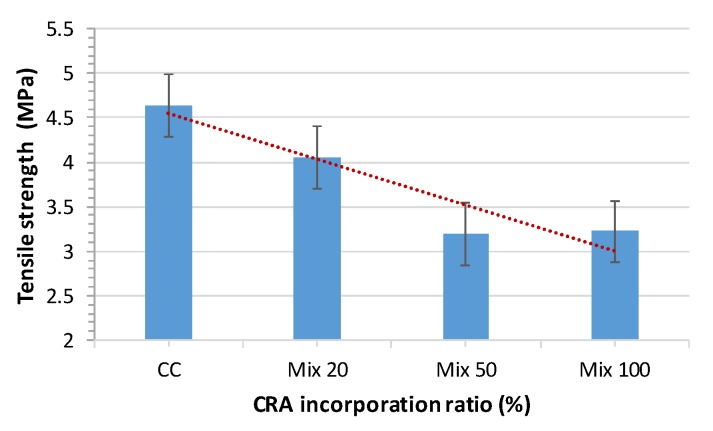
28-day tensile strength test results.

In terms of splitting tensile strength, the results of all mixes are shown in Figure 15. Tensile strength was determined in the hardened state as per EN 12390-6:2010 [49] at 28 days, 300 × 150 mm cylindrical samples were used.

Tensile strength decreases with the replacement of CNA with CRA, as expected. For an increase in RCA content, the tensile strength decreased by 12.71%, 31.25% and 30.60% in the mixes with CRA ratios of 20%, 50% and 100% respectively.

Tuyan et al. [29] concluded that the tensile strength of CRA mixes is slightly lower than that of the control mix. This loss is attributed to the ITZ between the CRA and the old bonded mortar paste and to the characteristics of the recycled aggregates. The strength loss goes from 8.8% to 16%.

Modani et al. [30] concluded that, as the percentage of CRA increases, the tensile strength decreases. The fracture surface occurs in the ITZ between the CRA and the CNA.

**Figure 16:**
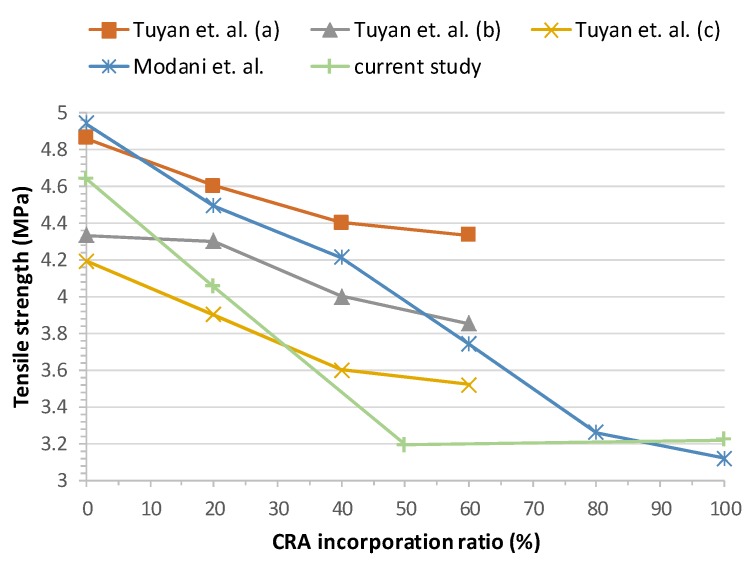
28-day splitting tensile strength test results overview.

Concerning this parameter, our work matches previous results of other authors for substitution ratios between 20% and 50%, keeping the same trend. There is hardly a difference of ±1 MPa between them. Equally, for a 100% substitution, our results are in line with results obtained by Modani et al. [30]. Figure 16 shows the comparative results.

Other authors had similar test results and concluded that tensile strength decreases as the CRA incorporation ratio increases, which can be justified by the lower strength and greater porosity of CRA compared with CNA [21,27,29,30] (Figure 16). In our study, it is observed than the increase of CRA up to 20% led to a slight decrease of 13%, and the incorporation of 50% and 100% of CRA led to a similar effect with a decrease of 30%.

### 3.6. Flexural Strength

**Figure 17:**
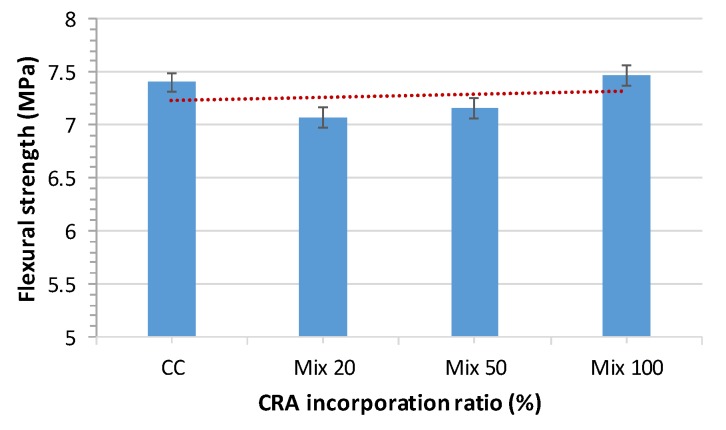
28-day flexural strength test results.

The results obtained for flexural strength for all mixes are shown graphically in Figure 17. Flexural strength was determined in the hardened state as per EN 12390-5:2009 [50] at 28 days, 100 × 100 × 400 mm prismatic samples were used.

In terms of flexural strength, the recycled aggregates studied herein did not lead to a notable difference relative to CC. The values are almost constant, and hardly suffer a variation of 4%.

Grdic et al. [28] concluded that flexural strength decreases with the increase of CRA. This reduction is due to the microstructural changes in the SCC. The reduction is 2.49% at SCC 50% CRA and 13.95% at SCC 100% CRA. The rest of the studies do not perform flexural tests.

**Figure 18:**
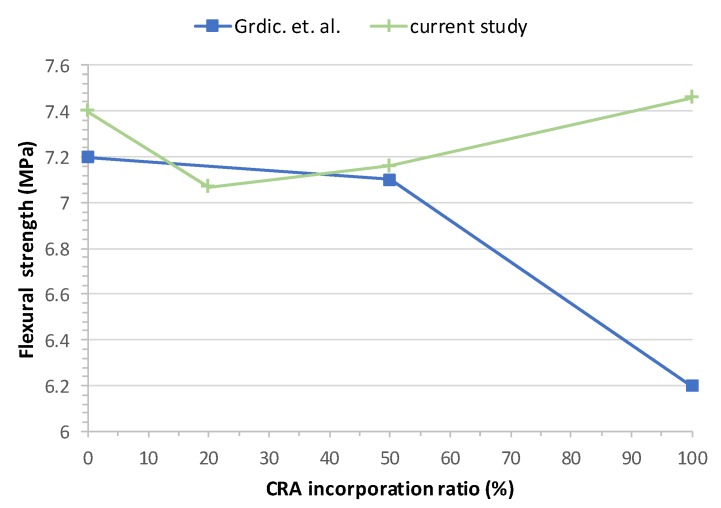
28-day flexural strength test overview.

Generally, authors found that flexural strength decreases as the replacement ratio increases, which is explained by the old mortar adhered to CRA [28] (Figure 18). In this case, the CRA used in the plant maintains its characteristics almost constant, has been washed and prepared and does not contain attached particles of mortar or gypsum, which explains this minimal variation in flexural strength.

Figure 18 shows a comparison between the study of Grdic et al. [28] and our work. For substitution ratios up to 50%, the results obtained are very similar, but for higher ratios, the results are very far apart. While our tests remain practically the same (even slightly higher than CC for a 100% substitution), the study of Grdic et al. [28] shows a strong decrease. As discussed above, this is probably due to the attached mortar in the recycled aggregates.

## 4. Conclusions

The following conclusions have been drawn from the development and results of this research study:

- It is feasible to achieve an SCC with recycled aggregates. SCC was produced with this C&DW as aggregate in the proportions recommended by EHE-08 of 20% substitution and above 50% and 100% with minimal loss in the properties. The SP content needs to be increased as RCA content increases, so CC concrete contains 0.8% of the weight cement, Mix 20 contains 1%, Mix 50 contains 1.2%, and Mix 100 contains 1.35%,
- The aggregates studied showed characteristics within the limits established by EHE-08 for recycled aggregates, a large quantity of concrete (71%) and natural stone (27%) in composition and a low proportion of ceramic, which means absorption is not penalized and it is within the limits allowed by the standard,
- The consistency of recycled aggregate mixes is not significantly affected by adding coarse recycled aggregates and it shows adequate workability. The slump flow increases with the CRA incorporation ratio due to the gradual increase in SP content,
- The compressive strength is excellent and coherent with the composition used when producing concrete and is completely feasible for use in structural elements. The compressive strength increases with the CRA incorporation ratio. Mix 20 has 20% more strength than CC and Mix 50 about 18%. Only the total replacement mix, Mix 100, suffered a small drop in compressive strength, 5% less than the CC,
- Tensile strength decreases with the replacement of CNA with CRA by 12.71%, 31.25% and 30.60% in the mixes with CRA ratios 20%, 50% and 100% respectively. Regarding flexural strength, recycled aggregates concrete mixes did not show a notable difference with respect to CC. The values are almost constant, and hardly suffer a variation of 4%,
- The good self-compactability, mechanical resistance and durability results obtained should be an incentive to reconsider the use of this type of waste as recycled aggregates in concrete, which would also help make the construction process more sustainable.

The following could be considered as future lines of research for this study:

- Readjusting the percentage of SP in some of the mixes, especially in Mix 20,
- Partial or total replacement of FRA (fine recycled aggregates) in the mixes,
- Microscopy and durability studies of the resulting concrete.

The results of this research show that coarse recycled aggregates may be considered feasible and workable for use in SCC as appropriate mechanical properties can be achieved with contents that are completely satisfactory for structural purposes. Their use would be a significant contribution to the sustainability of the construction sector.

## Figures and Tables

**Table 1:** Portland cement characteristics.

**Chemical Composition**	**Value (wt.%)**	**Limit (wt.%)** [39]
Clinker (SiO_2_, Fe_2_O_3_, Al_2_O_3_, CaO, MgO and SO_3_)	54	35–64
Blast-furnace slag	41	36–65
Minor components	5	≤5
LOI (Loss on ignition)	1.5	≤5
**Physical Characteristics**		
Le Chatelier (mm)	0.1	≤5
Setting time initial (min)	210	≥60
Setting time final (min)	260	≥60
**Mechanical Characteristics**		
Compressive strength (MPa) 2 days	20.1	≥13.5
Compressive strength (MPa) 28 days	56.6	≤42.5 and ≤62.5

**Table 2:** Chemical composition: natural lime filler, coarse natural and fine natural aggregates

Chemical Composition	Filler Value (wt.%)	CNA ^1^ Value (wt.%)	FNA ^2^ Value (wt.%)
SiO_2_	6%	98.09%	95.31%
Al_2_O_3_	1.2%	1.17%	2.24%
Fe_2_O_3_	0.69%	0.27%	1.06%
CaO	52.5%	0.05%	0.16%
MgO	1.4%	-	-
K_2_O	-	0.14%	0.38%
TiO_2_	-	-	0.17%
MnO	-	-	0.05%
CuO	-	-	0.03%
ZrO_2_	-	-	0.03%
LOI (Loss on ignition)	38.21%	0.28%	0.57%

^1^ CNA = coarse natural aggregates, ^2^ FNA = fine natural aggregates.

**Table 3:** Composition and physical and mechanical characterization of the recycled aggregates.

Parameter	Standard	Value	Limit EHE-08 [31]
Composition (%)	EN 933-1:2012 [41]		
Fl (floating particles) (%)		0	≤1
X (gypsum and impurities) (%)		0.04	≤1
Rc (concrete) (%)		70.7	-
Ru (natural stone) (%)		27	-
Rb (bricks and tiles) (%)		2.3	≤5
Ra (bituminous mat.) (%)		0	≤1
Rg (glass) (%)		0	≤1
Flakiness index (%)	EN 933-3:2012 [42]	5.7	≤35
Density and absorption	EN 1097-6:2014 [43]		
P_a_ (apparent density) (Mg/m^3^)		2.52	-
P_od_ (oven-dry density) (Mg/m^3^)		1.94	-
P_ssd_ (saturated surface dry density) (Mg/m^3^)		2.17	-
WA^24^ (%)		4.8	≤5
Los Angeles coefficient (%)	EN 1097-2:2010 [44]	36	≤40

**Table 4:** Physical and mechanical characterization of the coarse natural aggregates.

Parameter	Standard	Value	Limit EHE-08 [31]
Flakiness index (%)	EN 933-3:2012 [42]	3.6	≤ 35
Density and absorption	EN 1097-6:2014 [43]		
P_a_ (apparent density) (Mg/m^3^)		3.01	-
P_rd_ (oven-dry density) (Mg/m^3^)		2.66	-
P_ssd_ (saturated surface dry density) (Mg/m^3^)		2.78	-
WA^24^ (%)		4.3	≤ 5
Los Angeles coefficient (%)	EN 1097-2:2010 [44]	34	≤ 40

**Table 5:** Proportion of natural and recycled aggregates in the mixes.

Mixes	Coarse Natural Aggregate (%)	Coarse Recycled Aggregate (%)
CC ^1^	100%	0%
Mix 20 ^2^	80%	20%
Mix 50 ^3^	50%	50%
Mix 100 ^4^	0%	100%

^1^ CC = control concrete, ^2^ Mix 20 = mix 20% coarse recycled aggregates, ^3^ Mix 50 = mix 50% coarse recycled aggregates, ^4^ Mix 100 = mix 100% coarse recycled aggregates.

**Table 6:** Mix proportion (kg/m^3^).

Content (m^3^)	CC ^1^	Mix 20 ^2^	Mix 50 ^3^	Mix 100 ^4^
Cement (kg)	400	400	400	400
Lime filler (kg)	58	58	58	58
Water (kg)	190	190	190	190
Natural sand (kg)	904	904	904	904
Coarse natural aggregate (kg)	700	560	350	0
Coarse recycled aggregate (kg)	0	140	350	700
Superplasticizer (% weight of cement)	0.8	1	1.2	1.35
W/C ratio	0.47	0.47	0.47	0.47

^1^ CC = control concrete, ^2^ Mix 20 = mix 20% coarse recycled aggregates, ^3^ Mix 50 = mix 50% coarse recycled aggregates, ^4^ Mix 100 = mix 100% coarse recycled aggregates.

**Table 7:** Self-compactability of the samples designed.

Mix				Flow	J-Ring
	T_500_ (s) ^1^	Limit EHE-08 [31]	SF (mm) ^2^	Limit EHE-08 [31]	PJ (mm) ^3^
CC	4 4	≤8 ≤8	600 600	550 ≤ SF ≤ 850 550 ≤ SF ≤ 850	14 14
Mix 20	5	≤8	590	550 ≤ SF ≤ 850	15
	5	≤8	580	550 ≤ SF ≤ 850	10.25
Mix 50	5	≤8	770	550 ≤ SF ≤ 850	11
	5	≤8	780	550 ≤ SF ≤ 850	5.3
Mix 100	4	≤8	730	550 ≤ SF ≤ 850	5.6
	4	≤8	770	550 ≤ SF ≤ 850	5.5

^1^**T_500_** = Time until the concrete reaches the 500 mm circle. ^2^ **SF** = flow, ^3^ **PJ** = passage capacity calculated using the blocking scale.
